# Bio-organic fertilizer with reduced rates of chemical fertilization improves soil fertility and enhances tomato yield and quality

**DOI:** 10.1038/s41598-019-56954-2

**Published:** 2020-01-13

**Authors:** Lin Ye, Xia Zhao, Encai Bao, Jianshe Li, Zhirong Zou, Kai Cao

**Affiliations:** 1 Horticulture College, Northwest A&F University, Yangling, Shanxi China; 2 Agriculture College, Ningxia University, Yinchuan, Ningxia China; 3 The Agriculture Ministry Key Laboratory of Agricultural Engineering in the Middle and Lower Reaches of Yangtze River, Institute of Agricultural Facilities and Equipment, Jiangsu Academy of Agricultural Sciences, Nanjing, China

**Keywords:** Ecology, Plant physiology

## Abstract

The extensive use of chemical fertilizers poses serious collateral problems such as environmental pollution, pest resistance development and food safety decline. Researches focused on applying plant-beneficial microorganisms to partially replace chemical fertilizer use is increasing due to the requirement of sustainable agriculture development. Thus to investigate the possibility of a plant-beneficial *Trichoderma* strain and its bio-organic fertilizer product in saving chemical fertilizer application and in improving crop quality, a field trial and continuous pot experiments were carried out with tomato. Four treatments were set up: a reduced application of chemical fertilizer (75% of the conventional application) plus *Trichoderma*-enriched bio-organic fertilizer (BF), organic fertilizer (OF) or *Trichoderma* spore suspension (SS), with using the 100% rate of the conventional chemical fertilizer as the control (CF). The results showed that the total soluble sugar, Vitamin C and nitrate accumulations were, respectively, +up to 24%, +up to 57% and –up to 62% in the tomatoes of the BF treatment compared to those of the control (CF). And both of the pot and field trials revealed that reduced rates of chemical fertilizer plus bio-organic fertilizer produced tomato yields equivalent to those obtained using the 100% of the chemical fertilizer. However, application with the inoculant alone (SS) or combined with the organic fertilizer alone (OF) would lead to a yield decreases of 6–38% and 9–35% over the control. Since the increased abundance of soil microflora and the enhanced soil fertility frequently showed positive linear correlations especially in the BF-treated soils, we conclude that the efficacy of this bio-organic fertilizer for maintaining a stable tomato yield and improving tomato quality may be due to the improved soil microbial activity. Thus, the results suggest that the *Trichoderma* bio-organic fertilizer could be employed in combination with the appropriate rates of chemical fertilizers to get maximum benefits regarding yield, quality and fertilizer savings.

## Introduction

Application of chemical fertilizers is one of the most adopted regimes in developing intensive agriculture nowdays^1,2^. However, the continuous long-term use of chemical fertilizer has led to many unexpected effects. For example, the productivity-cost does not scale linearly and results in a huge waste of mineral resources. In addition, millions of tons of synthetic nutrients that are loaded to soil yearly are not absorbed by plants. About up to 50% of N and 90% of P have been reported to run off from crop fields and escape into the atmosphere or water sources, thereby causing the generation of greenhouse gas, eutrophication in aquatic system and salinization in soil^2,3^. Besides, the excessive application of chemical fertilizer leads to food safety and quality decline problems, such as nitrate accumulation in vegetal products. Indeed, several studies have demonstrated that organic farming, which strictly prohibits synthetic fertilizers, provides an alternative that has the potential to minimize the negative influence from by using chemical fertilization, and the products from the organic farming systems are generally endowed with improved nutritional properties^4–7^. For example, the work of Caris-Veyrat *et al*.^4^ reported that organic tomatoes had higher contents of carotenoids, polyphenols and Vitamin C than the ones from the conventional farming agriculture. However, organic farming is always associated with lower yield of crops and thus a higher cost. Therefore, the use of chemical fertilizers is not able to be eliminated once a considerable food production is expected^1,8^. Another current proposition for solving the agro-environmental problem is the integrated nutrient management that does not aim to entirely remove synthetic fertilizers in the near future instead suggests using microbial inoculations to reduce the amount of fertilizers applied^1^. Previous studies have shown that the inefficiency of synthetic fertilizers can be alleviated by applying plant growth-promoting microbes (PGPM), which are capable of increasing nutrient use efficiency and plant growth while reducing fertilizer inputs by up to 50% without causing any yield loss relative to the fully fertilized controls^2,9,10^.

PGPM exert considerable beneficial effects on the growth and development of plants. Many microbial genera have been commercially applied in agriculture^11,12^. For example, the plant-beneficial effect of *Trichoderma harzianum* has been widely studied and some of the strains are commercially applied to fields in China^12,13^. They have been previously shown with significant effects on plant growth, root development, biocontrol of soil-borne diseases and inducing plant systemic resistance^12–14^. To effectively apply such PGPM, it was suggested to enrich the strain in some organic substrate (e.g., as the form of bio-organic fertilizer) and then apply, since *T*. *harzianum* was reported to survive better in the rhizosphere after enriched in organic substrates^13,15,16^. For nearly all commercial uses of *Trichoderma* for biocontrol and plant growth and yield enhancement, a better understanding of the interactions among the *Trichoderma*, the fertilizer, the soil and the plant is essential. Although the number of research papers focused on reducing the inputs of fertilizer through different PGPM inoculations has increased in recent years, there are few reports concerning the combination of *Trichoderma*-enriched bio-organic fertilizer and reduced chemical fertilizer, especially in a long-term follow-up study, or concerning its potential effect on food quality^16–18^. Until recently, the focus has primarily been on the yield rather than the gustative or micro nutritional quality of fresh vegetal products. It could be acceptable for staple foods, but, as far as vegetables and fruits have been concerned, it can be argued the food quality matters more than the energy supply^4^. Tomato (*Solanum lycopersicum*), one of the most popular vegetables worldwide, is cultivated in over 140 countries^19^. It has many metabolites responsible for health and nutritional values, thus making it as an excellent model for studying fruit development and food safety^20^. However, in spite of the importance of vegetable quality to human health, there has been little improvement in the tomato fruit quality since producers preferentially select cultivars with traits of resistance to biotic and abiotic stress, extended shelf-life and appearance^19^. And the understanding of how fertilization regimes influence tomato quality stay poor.

Therefore, the aims of the present research were to investigate 1) if PGPM inoculants (*Trichoderma* or *Trichoderma* bio-organic fertilizer) combined with reduced rates of chemical fertilizer would produce tomato yield and fruit quality equivalent to those obtained using full rates of chemical fertilizer, and 2) how the soil fertility responses to these fertilization regimes in a continuous cropping system. We therefore investigated the effectiveness of a *Trichoderma* bio-organic fertilizer on tomato yield and quality in an open field and evaluated the effect of *Trichoderma* inoculation on some chemical and biological properties of the soil related to plant growth via continuous 4-season pot experiments. Due to the importance of soluble sugars to a tomato’s organoleptic quality as well as that of Vitamin C (Vc) to nutrition and nitrate accumulation to safety, it is acceptable to use these three indexes to indicate the quality of a tomato fruit^7,19,21,22^. The introduction of a PGPM agent and fertilization could influence the soil’s microbial diversity and nutrient status and could subsequently change the soil quality; thus, the soil microbial population and the soil total and available nutrients were measured at the end of each growing season. In addition, strains with the potential of promoting plant growth and providing other benefits must be able to colonize roots^23^; therefore, the number of *Trichoderma* in the rhizosphere soil was determined in this study.

## Results

### Tomato yield

The results (Table 1) of both field trials and pot experiments indicated that the tomato yields from treatments with 75% chemical fertilizer plus bio-organic fertilizer (BF) were statistically equivalent to the yields obtained using full rate of chemical fertilizer (CF) without inoculants, while the yields obtained using 75% chemical fertilizer with *Trichoderma* spore suspension (SS) or organic fertilizer (OF) were less than those of the CF treatment in most cases significantly (*P* < 0.05) (Table 1). In contrast to the control (CF), the OF and SS treatments led to a decrease of 6–38% and 9–35%, respectively, in tomato yield in the pot experiments. The results were slightly different among the 4 pot experiments as the yields of the 4 treatments in the 1st season did not differ from each other. Because the data of the 1st field trial were more persuasive than those from the 2nd trial, here and similarly hereafter, only the data of the 1st field trial are shown.

**Table 1:** Effect of different treatments on tomato yield in pot experiments and field trials.

Treatments^a^	Pot Experiments (kg pot^−1^)				Field Trial
	1st Season	2nd Season	3rd Season	4th Season	(kg plot^−1^)
CF	0.74 ± 0.06a	0.22 ± 0.01b	0.55 ± 0.01a	0.23 ± 0.01a	82.85 ± 3.71ab
BF	0.77 ± 0.13a	0.28 ± 0.02a	0.55 ± 0.01ab	0.25 ± 0.01a	86.78 ± 8.76a
OF	0.68 ± 0.05a	0.16 ± 0.01c	0.52 ± 0.01b	0.18 ± 0.00b	70.51 ± 6.76b
SS	0.68 ± 0.12a	0.18 ± 0.01c	0.44 ± 0.03c	0.17 ± 0.01b	—

^a^CF: 100% chemical fertilizer; BF: 75% chemical fertilizer + bio-organic fertilizer; OF: 75% chemical fertilizer + organic fertilizer; SS: 75% chemical fertilizer + spore suspension. The mean value ± standard deviation (n = 5). Values with the same letter do not differ significantly (*P* < 0.05).

### Tomato quality parameters

**Figure 1:**
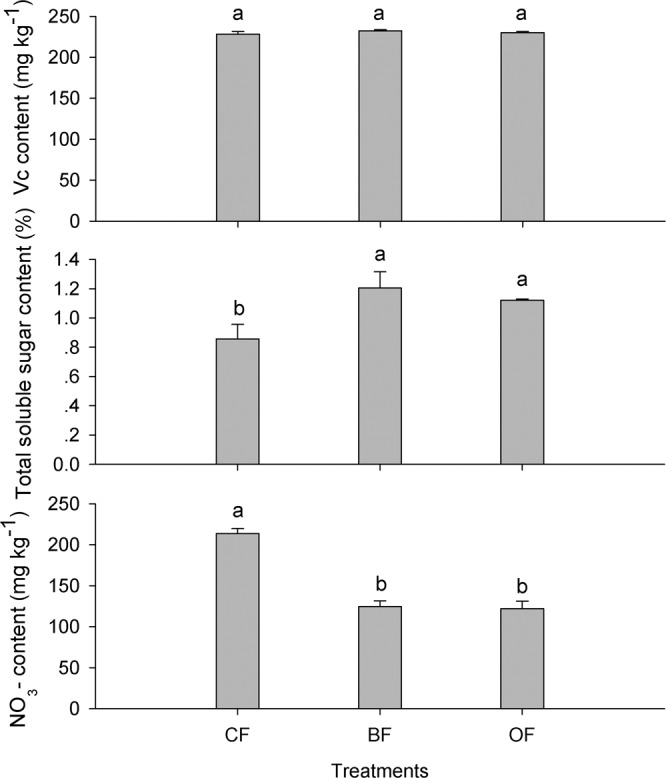
Effects of different treatments on tomato fruit quality in the open field. CF: 100% chemical fertilizer; BF: 75% chemical fertilizer + bio-organic fertilizer; OF: 75% chemical fertilizer + organic fertilizer. Error bars represent the standard deviation calculated from 5 replicates. Bars followed by the same letter are not significantly different at *P* < 0.05.

**Figure 2:**
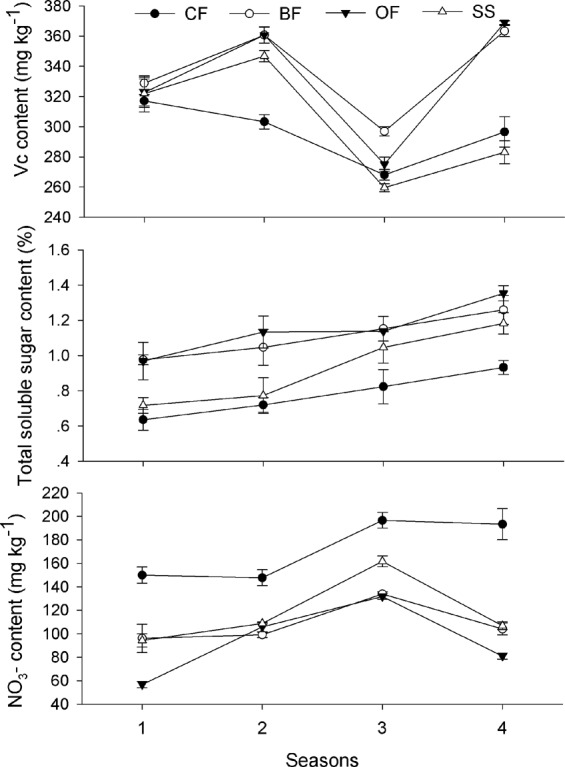
Effects of different treatments on tomato fruit quality with cropping seasons in the greenhouse. CF: 100% chemical fertilizer; BF: 75% chemical fertilizer + bio-organic fertilizer; OF: 75% chemical fertilizer + organic fertilizer; SS: 75% chemical fertilizer + spore suspension. Error bars represent the standard deviation calculated from 5 replicates.

The results in Fig. 1 showed that, in field trials, the fertilization treatments had significant (*P* < 0.05) effect on the total soluble sugars (TSS) content and NO_3_- accumulation in tomato fruit while had little effect on Vc content. Specifically, TSS content was approximately 40% higher in fruit from the BF or OF treatments than it was in fruit from the control (CF), whereas the NO_3_- content in fruit from the CF treatment was approximately 70% higher than that in fruit from the reduced chemical fertilization treatments (BF and OF). The results in Fig. 2 showed that different fertilization significantly affected the quality of the tomato fruit in the pot experiments. The variance in the Vc content observed in the tomato fruit in the 4 treatments was not obvious in the 1st and 3rd seasons, but it was evident in the 4th season: specifically, the Vc contents in the organic material added treatments (BF and OF) were 23–30% higher than the Vc contents in the synthetic fertilizer treatments (CF and SS) (Fig. 2a). The chromatograms of Vc and NO_3_- analysis are illustrated with Figs. S1 and S2. The TSS contents in the four treatments gradually increased with continuous cropping, and the NO_3_- contents generally increased first and subsequently decreased; the TSS content in the 3 reduced chemical fertilizer treatments (BF, OF and SS) were much higher than with the full rate chemical fertilizer treatment (CF), while the trend was opposite in NO_3_- accumulation throughout the 4 cropping seasons (Fig. 2b,c).

### Soil nutrients content and organic matter

**Figure 3:**
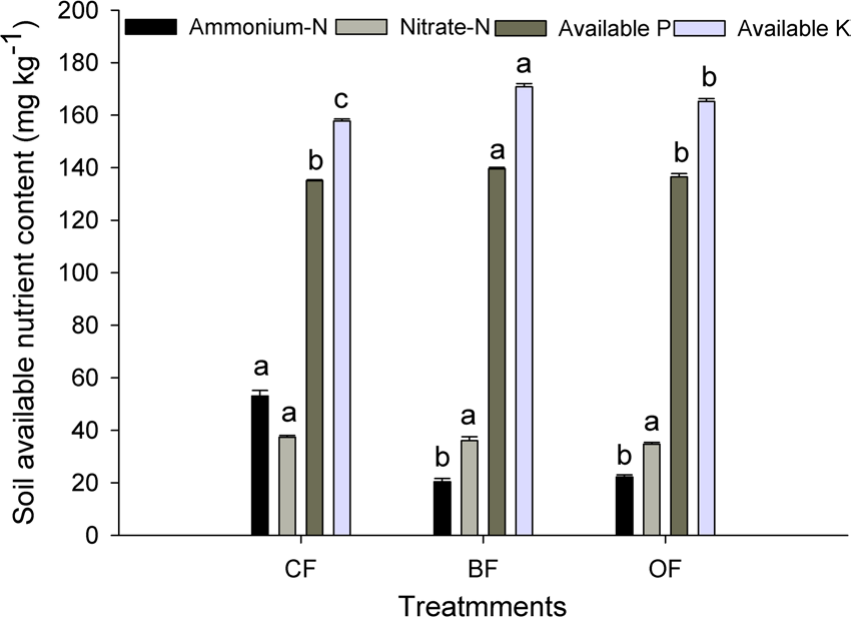
Effects of different treatments on soil available nutrients in the open field. CF: 100% chemical fertilizer; BF: 75% chemical fertilizer + bio-organic fertilizer; OF: 75% chemical fertilizer + organic fertilizer. Error bars represent the standard deviation calculated from 5 replicates. Bars followed by the same letter are not significantly different at *P* < 0.05.

In general, the soil available nutrient contents at the end of the growing season reflect the soil nutrient supply. In the field trial, the available P and available K in the BF treatment were significantly higher than the contents in CF and OF treatments (*P* < 0.05) (Fig. 3). Conversely, no significant differences were observed in soil nitrate-N among the 3 treatments, and the ammonium-N in the CF treatment was markedly higher than the contents in the BF and OF treatments.

**Figure 4:**
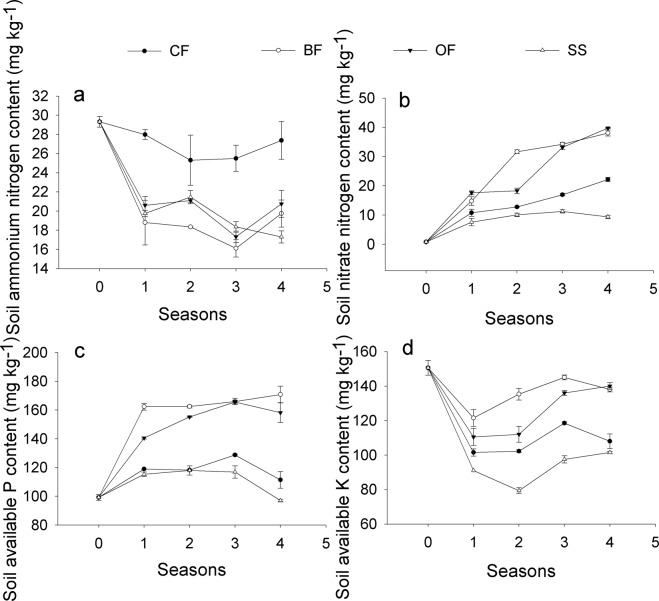
Effects of different treatments on soil available nutrients with cropping seasons in the greenhouse. (**a**) Soil ammonium-N; (**b**) Soil nitrate-N; (**c**) Soil available P; (**d**) Soil available K. CF: 100% chemical fertilizer; BF: 75% chemical fertilizer + bio-organic fertilizer; OF: 75% chemical fertilizer + organic fertilizer; SS: 75% chemical fertilizer + spore suspension. Error bars represent the standard deviation calculated from 3 replicates.

Figure 4 showed the fluctuations in available nutrients over time in the pot experiments. A similar trend of available P and available K was found in both the pot experiments and the field trials. The available P and available K in the organic material added treatments (BF and OF) were significantly higher than the contents in the synthetic fertilizer treatments (CF and SS), while the ammonium-N in the CF treatment was the highest among the 4 treatments throughout the 4 copping seasons (Fig. 4a,c,d). The soil nitrate-N gradually grew with time, and its content in the BF and OF treatments remained at a higher level compared with that of the CF (Fig. 4b). Higher levels of nitrate-N, available P and available K were more frequently observed in the BF treatment, whereas the lowest contents of available nutrients, including all of the above indexes, were mostly found in the SS treatment.

**Figure 5:**
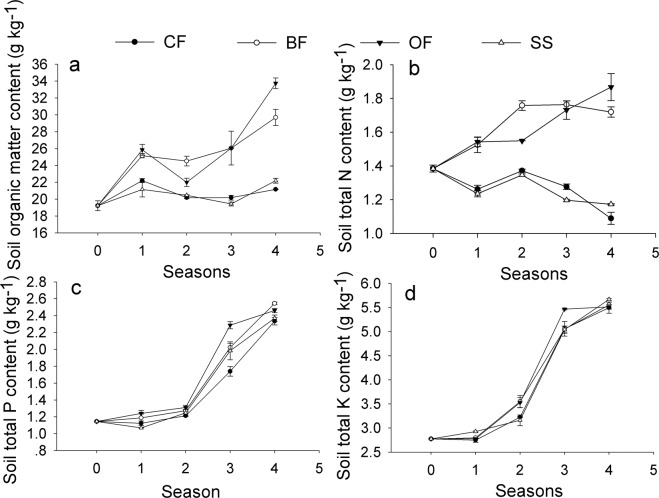
Effects of different treatments on soil total nutrients with cropping seasons in the greenhouse. (**a**) Soil organic matter; (**b**) Soil total N; (**c**) Soil total P; (**d**) Soil total K. CF: 100% chemical fertilizer; BF: 75% chemical fertilizer + bio-organic fertilizer; OF: 75% chemical fertilizer + organic fertilizer; SS: 75% chemical fertilizer + spore suspension. The standard deviation (n = 3) was analyzed using a one-way ANOVA.

The data in Fig. 5 showed that bio-organic or organic fertilization (BF and OF) significantly enriched soil fertility, as the soil organic matter increased by 55–75%, total N by 25–36%, total P by 116–123% and total K by 99%–100% after the 4-season fertilization. Total P and total K also improved following the CF and SS treatments (Fig. 5c,d), while the soil organic matter maintained a stable level, and the total N decreased with time in these 2 treatments (Fig. 5a,b). Higher levels of total nutrients were always found in the OF treatment.

### Soil bacteria, fungi, actinomycetes and *Trichoderma* population

**Figure 6:**
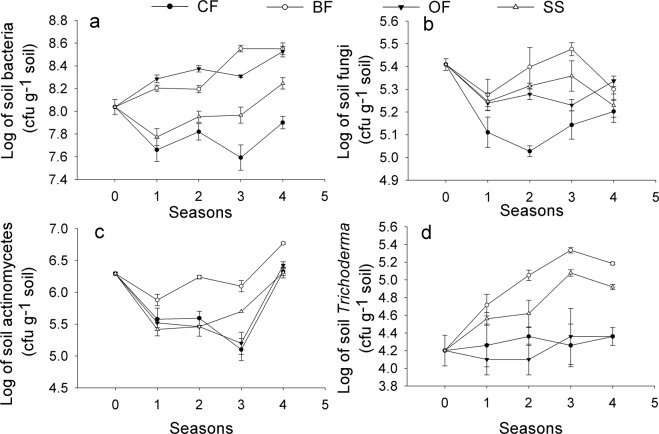
Effects of different treatments on microbial abundance with cropping seasons in the greenhouse. (**a**) Total population of cultivable bacteria in pot soil; (**b**) Total population of cultivable fungi in pot soil; (**c**) Total population of cultivable actinomycetes in pot soil; (**d**) Total population of cultivable *Trichoderma* in pot soil. CF: 100% chemical fertilizer; BF: 75% chemical fertilizer + bio-organic fertilizer; OF: 75% chemical fertilizer + organic fertilizer; SS: 75% chemical fertilizer + spore suspension. Quantification of the total cultivable microorganisms in the soils was performed using the standard 10-fold dilution plating method via colony forming units (cfu) and expressed as its logarithm. The data fulfilled the prerequisites of one-way ANOVA.

The colony forming unites (cfu) of culturable soil microbes including bacteria, fungi and actinomycetes and *Trichoderma*, were transformed into their logarithms, as shown in Fig. 6. The total bacteria population of the pot soils in the BF, OF and SS treatments increased (to Log8.6, Log8.5 and Log8.2, respectively), while the number in the CF treatment decreased (to Log7.9) slightly (Fig. 6a). The total fungi population generally first decreased, then increased and divided into 2 groups (one group including the BF and OF treatments decreased, and the other including the CF and SS treatments kept increasing) (Fig. 6b). The total actinomycetes population for all of the treatments first showed a decrease and then an increase (Fig. 6c). The *Trichoderma* population in the soil generally first increased and then decreased in the BF and SS treatments, whereas it remained at a low level in the CF and OF treatments, which were non-inoculated (Fig. 6d). Additionally, the microbial population in the BF treatment always maintained the highest level among the 4 treatments, while the CF treatment was more frequently found to have the lowest microbial population.

### Pearson correlations of soil nutrients and microbial abundance with tomato yield and quality

In total, 120 correlations among the soil nutrients, microflora, tomato yield and fruit quality properties were detected (Table 2). Of these correlations, 19 and 28 correlations had a significance level of 0.05 and 0.01, respectively. In particular, there was no correlation between the tomato fruit yield and the other parameters detected. The Vc content in the tomato fruit was found to have a positive correlation with soil organic matter but a negative correlation with NO_3_- accumulation in fruit. The TSS content in the fruit was positively correlated with all of the soil parameters detected in this work, with the exception of a negative correlation with the soil ammonium-N. The NO_3_- content in the fruit was negatively correlated with several soil parameters, including total N, organic matter and the bacteria population, and positively correlated with ammonium-N. The *Trichoderma* population in soil showed positive internal links with the soil fungi and actinomycetes populations, as well as a negative link with the soil ammonium-N. Furthermore, 2 major correlation groups with numerous internal links were observed. The first group consisted of strong positive links among the soil nutrient parameters themselves, especially the strong positive links between the organic matter and the other nutrient parameters. The second group included connections (some positive and some negative) among the soil nutrient parameters and the soil microflora.

**Table 2:** Pearson correlations of soil nutrients and microbial abundance with tomato yield and quality.

	TN	TP	TK	OM	AN	NN	AP	AK	Bac	Fun	Act	Tri	Yield	Vc	TSS	NO_3_-
TN	1															
TP	0.188	1														
TK	0.115	0.967^**^	1													
OM	0.821^**^	0.463	0.309	1												
AN	−0.496	−0.233	−0.216	−0.352	1											
NN	0.836^**^	0.590^*^	0.533^*^	0.817^**^	0.305	1										
AP	0.920^**^	0150	0.091	0.716^**^	−0.447	0.795^**^	1									
AK	0.825^**^	0.483	0.457	0.762^**^	−0.367	0.913^**^	0.847^**^	1								
Bac	0.777^**^	0.478	0.358	0.772^**^	−0.683^**^	0.714^**^	0.712^**^	0.677^**^	1							
Fun	0.516^*^	0.247	0.236	0.398	−0.733^**^	0.480	0.504^*^	0.423	0.647^**^	1						
Act	0.282	0.569^*^	0.450	0.551^*^	−0.202	0.522^*^	0.221	0.432	0.556^*^	0.401	1					
Tri	0.198	0.327	0.334	0.138	−0.616^*^	0.269	0.205	0.289	0.370	0.660^**^	0.568^*^	1				
Yield	−0.069	−0.412	−0.423	−0.062	−0.001	−0.234	0.092	0.007	−0.282	−0.103	−0.438	−0.108	1			
Vc	0.534	−0.201	−0.327	0.525^*^	−0.042	0.350	0.462	0.201	0.430	0.280	0.392	−0.031	−0.304	1		
TSS	0.591^*^	0.669^**^	0.634^**^	0.699^**^	−0.591^*^	0.687^**^	0.537^*^	0.657^**^	0.879^**^	0.621^*^	0.661^**^	0.402	−0.433	0.281	1	
NO_3_-	−0.538^*^	0.214	0.360	−0.543^*^	0.545^*^	−0.173	−0.388	−0.144	−0.582^*^	−0.373	−0.211	−0.062	−0.007	−0.667^**^	−0.356	1

Abbreviations: TN, Soil total N; TP, Soil total P; TK, Soil total K; OM, Soil organic matter; AN, Soil ammonium-N; NN, Soil nitrate-N AP, Soil available P; AK, Soil available K; Bac, Soil bacteria population; Fun, Soil fungi population; Act, Soil actinomycetes population; Tri, Soil *Trichoderma* population; Yield, Yield of tomato; Vc, Vitamin C content of tomato fruit; TSS, Total soluble sugars content of tomato fruit; NO_3_-, NO_3_- content of tomato fruit. Correlations significant at the *P < 0.05 level and **P < 0.01 level (2-tailed).

## Discussion

The reduced load of fertilizers into crop fields without causing productivity lost is a feasible but difficult challenge^2^. The results of the field trials confirmed our hypothesis that the combination of *Trichoderma*-enriched bio-organic fertilizer with reduced (75%) chemical fertilizers (BF) could produce tomato yield equivalent to or higher than those obtained using full rates of the fertilizer (CF) (Table 1). This result was consistent with repeated observations in pot experiments. However, inoculation with the *Trichoderma* alone (SS, without organic substrate supply) or supplement with organic fertilizer alone (OF, without *Trichoderma*) would cause 6–38% and 9–35% decreases in yield over the control (CF) in the presence of 100% chemical fertilizer doses. Hence, only bio-organic fertilizer could be a viable supplementary strategy to maintain or increase tomato yields. Similar results regarding PGPM application was also reported by Adesemoye *et al*.^11^ that the growth and yield of tomato grown in a greenhouse with 75% fertilizer plus PGPM were statistically equivalent to those obtained using full fertilizer rates without PGPM. Moreover, some reports showed that bio-organic fertilizers could replace 23–52% of N fertilizer without any loss of yield^24^, which also indicating the potential role of bio-organic fertilizer in reducing the amount of chemical fertilizer load to soil. Whereas, few previously reported evidences regarding the influence of PGPM or its bio-fertilizer application on crop quality could be found^8,11^. The effects of the same treatment on the measured yield differed greatly throughout the four growing seasons may due to the variations of climatic conditions.

In this study, at reduced fertilizer rates (75%), inoculants consistently enhanced tomato fruit quality (Figs. 1 and 2). In particular, NO_3_- accumulation in fruit, which is associated with risks of several human health problems such as methemoglobinemia and gastric cancer^22^, from the reduced chemical fertilization (BF, OF and SS) was coupled with a lower (down to 62%) content compared with the full-rate fertilized control (CF) in the greenhouse. Actually, NO_3_- is relatively non-toxic, but its metabolite products, especially NO_2_-, raised concern in human health^25^. As 85–90% of an adult dietary intake of NO_3_- is from vegetables, while the vegetables tend to concentrate NO_3_- when grown in a N highly applied field^22,26^. Therefore, a proper control of the application rate of chemical fertilizers to soil would directly reduce our risks to some nitrate-related health problems. Moreover, the Vc and TSS contents in the tomato fruit in the treatments with added organic material (BF and OF) were significantly higher (up to 24% and 57%, respectively) than that of the control (CF). As suggested by Oliveira1 *et al*.^7^, organic farming creates stressing conditions for tomatoes and thus resulted in the higher accumulation of soluble solids in fruits such as sugars and other compounds that contribute to the nutritional quality of fruit, including Vc and phenolic compounds. The differences observed in tomato yield and quality for different treatments may originate from the differences in nitrogen availability because the organic fertilizers (including bio-organic fertilizer and organic fertilizer) release N (more obviously as ammonium-N in this study; Figs. 3 and 4) much more slowly than do chemical fertilizers, thus forcing the plants to struggle more for growth and resulting in a more complex diversion of protein synthesis and secondary metabolisms^4,27^.

Concerning the other elements, more available P and available K were found in the BF- and OF-treated soils at the end of each growing season. *T*. *harzianum* has been reported to be able to solubilize several plant nutrients^28^, and *T*. *asperellum* has been shown to enhance the P and Fe availability for cucumber once colonized on roots^29^. However, the enhancement of available K in the BF treatment in this study did not occur via K dissolution because T7 has shown no direct K dissolution ability *in vitro* under laboratory conditions (data not shown). Hence, the resulted enhancement of K availability must be due to alternative microbial effects. As shown in Fig. 6, the treatment with reduced chemical fertilization plus *Trichoderma* bio-organic fertilizer (BF) resulted in a greater increase of the population of bacteria, fungi and actinomycetes relative to the other treatments. Thus, the BF treatment was a more efficient tool to enhance the abundance of soil microflora, which had a potential effect on soil nutrient cycling. Based on the explanations by Adesemoye *et al*.^11^ and Huang *et al*.^30^, *T*. *harzianum* promotes plant root growth via the secretion of an auxin-like phytohormone. Better root growth impacts soil microbiome via secreting more exudates, like sugars and organic acids, and soil microbes, in turn, response with higher microbial abundance and frequent interactions leading to more available nutrients that affect plant growth and health. Gradually, better plant growth caused by more bio-available nutrients again stimulates more microbes moving towards the rhizosphere resulting in higher nutrient uptake by the inoculated plants. This process can be illustrated using Pearson correlations, which showed a sharp correlation of these two traits (soil nutrients and soil microflora), as in Table 2.

In addition, strong positive links between soil organic matter and the other soil nutrient parameters (e.g., total N, P and K contents) were observed in this continuous cropping system. The changes in soil organic matter based on the application of mature compost are important. It was suggested that the application of composts to soils in agriculture could provide long-term fertility and less off-site impacts^31^. However, the nutrient supply capability of the OF treatment was efficient as compared to that of the BF. Therefore, the combination of functional PGPM (i.e., T7) and organic fertilizer as the form of bio-organic fertilizer was the better choice to reduce the use of chemical fertilizers and promote tomato yield and quality. After fermentation in compost, T7 utilized the nutrients provided by the organic material and established themselves in it, which increased the viability of the added T7 and made these strains more competitive in the microbial community in rhizosphere^5^. This may also be the reason why the BF treatment was more effective in maintaining tomato yield compared with T7 alone (SS).

In conclusion, the results of the present work imply that the application of this *Trichoderma*-enriched bio-organic fertilizer rather than applying the PGPM inoculant alone could help to avoid the overuse of expensive chemical fertilizers to a significant extent without compromising the yield (at least in tomatoes). More importantly, the use of *Trichoderma*-enriched bio-organic fertilizer with lower fertilizer application leads to the enhancement of fruit quality and the improvement of soil fertility and microbial environment. There, bio-organic fertilizers like this could be employed to supplement to the reduced rate of chemical fertilizers, and should be further evaluated as an important component of the integrated nutrient management regime.

## Materials and Methods

### Microbial strain

*T*. *harzianum* strain T7 was maintained in our lab which was originally isolated from soil. It was cultured on potato dextrose agar (PDA) at 28 °C and stored at 4 °C on slants. The strain purity was confirmed by PCR and sequencing to exclude the contamination from bacteria and other fungi before use.

### Bio-organic fertilizer preparation

Spore suspensions of T7 were prepared by flooding PDA cultures of 7-day-old T7 with sterile water; the suspensions were then filtered through a double layer of sterile cheesecloth and adjusted to 10^6^ conidiospores per mL based on the hemocytometer counts. The bio-organic fertilizer used in this study was obtained by aerobically fermenting a mixed organic fertilizer with T7 spore suspension (100:1, w/v) for 7 days at < 50 °C. The mixed organic fertilizer was prepared from mature compost of pig manure, which contained 42.5% organic matter, 3.5% N, 2.1% P_2_O_5_, 1.2% K_2_O and 28.4% H_2_O. The final bio-organic fertilizer was stored at 4 °C and was used in the experiments only if the population of the T7 remained at the level of 10^6^ cfu per gram of dry matter.

### Field trials design

Field trials were conducted twice at a vegetable testing site in army horse ranch 6 team (located at longitude 106°27’ E and latitude 38°47’ N), Yinchuan, Ningxia Province, China. No specific permits were required for the described field studies. The location is not privately owned or protected, and the field studies did not involve endangered or protected species. The soil characteristics were pH 6.1, organic matter 27.6 g kg^−1^, ammonium-N 21.9 mg kg^−1^, nitrate-N 22.7 mg kg^−1^, available P 131.3 mg kg^−1^ and available K 218.7 mg kg^−1^. The above data were collected by measuring five soil samples from the field site before planting. The first crop season extended from August to November; the second crop season lasted from March to June. The field treatments were designed as (1) CF: 100% chemical fertilizer; (2) BF: 75% chemical fertilizer + bio-organic fertilizer; and (3) OF: 75% chemical fertilizer + organic fertilizer. The 100% chemical fertilizer contained 600 kg ha^−1^ YaraMila compound fertilizer (N: P: K = 15: 15: 15) and 300 kg ha^−1^ YaraLiva-Ca(NO_3_)_2_ fertilizer (N ≥ 15%, split into 3 × 100 kg, YARA international ASA, Oslo, Norway) and was applied in plots consistent with the local farmers’ fertilization practice. The 75% chemical fertilizer used in this study was based on the 100% fertilizer because a 75% rate was reported to be a threshold below which the PGPM-fertilizer interaction could not produce consistent nutrient uptake comparable to the non-inoculated full fertilizer rates^1,11^. The application rate of bio-organic fertilizer or organic fertilizer was 1800 kg ha^−1^. Each treatment with 5 plots was randomly arranged in the selected fields, and each plot (6.0 m × 1.6 m) contained 60 tomato seedlings. Conventional practices like watering (every ten days), scarifying and disinsection were equally given when needed.

### Pot experiments design

To investigate the effect of *T. harzianum* T7 inoculation alone (without organic substance) on tomato growth, yield and fruit quality and on the soil environment, another treatment (SS: 75% chemical fertilizer + spore suspension) was added to the pot experiments, and further experiments were conducted using the same treatments applied in the field trials. At planting, seedlings with CF treatment (100% chemical fertilizer) received 5.33 g plant^−1^ YaraMila compound fertilizer. They were then fertigated with 2.67 g plant^−1^ YaraLiva-Ca(NO_3_)_2_ between four and five weeks after planting. The reduced fertilization treatments (BF, OF and SS) received 75% of the chemical fertilizer and 50 g plant^−1^ bio-organic fertilizer, 50 g plant^−1^ organic fertilizer or 5 mL plant^−1^ *Trichoderma* spore suspension (10^6^ cfu mL^−1^). Each pot (30 × 30 × 35 cm^3^) contained 10 kg soil and 2 seedlings. The soil used in this work was a loam (pH 7.3) with an organic matter content of 19.2 g kg^−1^. The available P and K, ammonium-N and nitrate-N contents were 99.2, 150.5, 29.3 and 0.8 mg kg^−1^, respectively. Six pots were used for each treatment, and they were arranged randomly in the greenhouse, with a maintained temperature of 20–30 °C. The pot experiments were continuously carried out for four times with repeated fertilization and planting.

### Estimation of tomato yield and fruit quality parameters

Destructive harvest was conducted on the 100th day after planting in both field trials and pot experiments, and data regarding the tomato yields were collected. Ripe fruits were harvested 3 times manually from 60 plants for each plot in the field trials (10 plants for pot experiments), and 3 of the fruits were selected to form a replicate for each treatment.

The contents of Vc and nitrate (expressed in NO_3_-) in the tomato fruit were analyzed with an Agilent 1200 semi-preparative HPLC (Agilent Technologies, Santa Clara, Cal., USA). For the Vc content, 5 g of fruit samples were homogenized in 50 mL of ice-cold extraction buffer (5 g L^−1^ oxalic acid) for 15 min and then transferred to a 100 mL volumetric flask diluted with the extraction buffer. The homogenate was filtered through a 0.45 μm filter membrane. All of the procedures were performed at 4 °C. The mobile phase was composed of 0.05 M KH_2_PO_4_ solution and methanol at a ratio of 95:5 (v/v) and used at a flow rate of 1.0 mL min^−1^; the eluate was detected at 265 nm at room temperature. The standard curve of Vc was developed with ascorbic acid (Sigma, USA) from 5 mg L^−1^ to 100 mg L^−1^. To evaluate the nitrate content in the tomato fruit, 50 g of fresh fruit was homogenized in 50 mL of deionized water. Five grams of the homogenate was added to 50 mL of hot water (70–80 °C) and used for ultrasonication for 20 min. After cooling, 5 mL of the above extract was diluted to 25 mL with deionized water and filtered through a 0.45 μm filter membrane. The eluent was 0.03 M KH_2_PO_4_-H_3_PO_4_ buffer (pH = 3.3) at a 1 mL min^−1^ flow at room temperature at 210 nm. Standard control was set up from 0.1 mg L^−1^ to 10 mg L^−1^ with KNO_3_. The TSS content was measured using an anthrone colorimetric method at 620 nm as described by Allen^32^.

### Soil sampling and nutrient content analysis

A composite soil sample (with three replicates) was collected from the pot soil which was mixed thoroughly manually before splitting into pots and was referred to as the 0th soil sample. At every harvest, soil samples were collected from the harvested plant roots of each treatment as described by Hervás *et al*.^33^ and referred to as the 1st, 2nd, 3rd and 4th soil samples. The tomato plants were carefully removed from the pots and gently shaken to detach the soil. Soils still adhering to the roots were defined as the rhizosphere soil samples. The soil samples for the field trials were prepared at harvest as described above for the pot experiment sampling.

The total N content and organic matter of soil samples were analyzed using a Vario EL elemental analyzer (Elementar Analysensysteme GmbH, Hanau, Germany). The total P and total K were analyzed using inductively coupled plasma-atomic emission spectrometer (Agilent 710 ICP-OES, Agilent Technologies, Santa Clara, Cal., USA). The soil available nutrients, including soil available P and available K, were determined following Shen *et al*.^34^, and the soil available N (ammonium-N and nitrate-N) was analyzed using a continuous-flow analyzer (AutoAnalyzer 3, Bran + Luebbe GmbH, Germany).

### Soil cultivable microbial count

The populations of soil cultivable bacteria, fungi, actinomycetes and *Trichoderma* were detected using the standard 10-fold dilution plating method. Specifically, Beef extract medium was used for the characterization of total bacteria population, Martin medium for fungi, Gause NO.1 medium for actinomycetes^35^ and a modified *Trichoderma* selective medium [1 L contained water, 800 mL; agar, 20 g; glucose, 3 g; NH_4_NO_3_, 1 g; KH_2_PO_4_, 0.9 g; MgSO_4_·7H_2_O 0.2 g; KCl, 0.15 g; rose Bengal, 0.15 g; chloramphenicol, 0.25 g; streptomycin, 0.05 g; quintozene (PCNB), 0.15 g; Captan, 0.1 g; propamocarb (772 g of active ingredient per liter), 1.2 mL; and Triton X-100, 1 mL] based on TSM medium^36^ for *Trichoderma* population. The data were expressed as the number of cfu per gram dry soil.

### Statistical analysis

The results were expressed as the means ± standard deviations and were calculated and statistically examined using an analysis of variance and a Duncan’s multiple range test. Statistical significance was considered at *P* < 0.05, unless otherwise stated. Most tests were conducted using SPSS version 13.0 (SPSS, Inc., Chicago, IL, USA). The Pearson correlation coefficient of pairwise correlation analysis was performed using SPSS to relate the tomato yield, quality and soil chemical and biological properties.

## Supplementary information

Supplementary file.
