# Supplementary material for: Bio-organic fertilizer with reduced rates of chemical fertilization improves soil fertility and enhances tomato yield and quality
Source: Sci Rep. 2020 Jan 13;10:177. doi: 10.1038/s41598-019-56954-2 (PMC6957517; doi:10.1038/s41598-019-56954-2)
Supplement: Supplementary file 1 — Supplementary file. [file 41598_2019_56954_MOESM1_ESM.docx]

**Bio-organic fertilizer with reduced rates of chemical fertilization improves soil fertility and enhances tomato yield and quality**

Lin Ye^1, 2*^, Xia Zhao^1, 2^, Encai Bao^3^, Jianshe Li^2^, Zhirong Zou^1*^, Kai Cao^3^

1 Horticulture College, Northwest A&F University, Yangling, Shanxi, China

2 Agriculture College, Ningxia University, Yinchuan, Ningxia, China

3 The Agriculture Ministry Key Laboratory of Agricultural Engineering in the Middle and Lower Reaches of Yangtze River, Institute of Agricultural Facilities and Equipment, Jiangsu Academy of Agricultural Sciences, Nanjing, China

^*^Corresponding authors email address: [yelin.3993@163.com;](mailto:yelin.3993@163.com;) zouzhirong2005@hotmail.com

**Supplementary files**

**
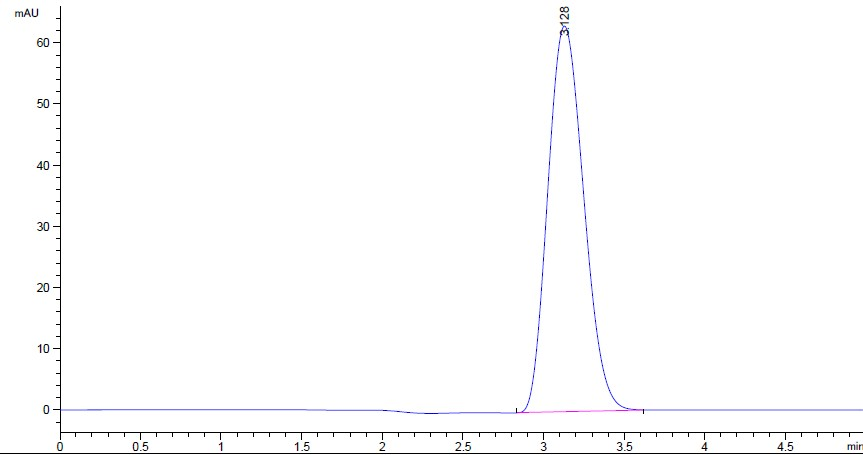
**

**Supplementary file S1** The chromatogram of Vc analysis for tomato fruit.


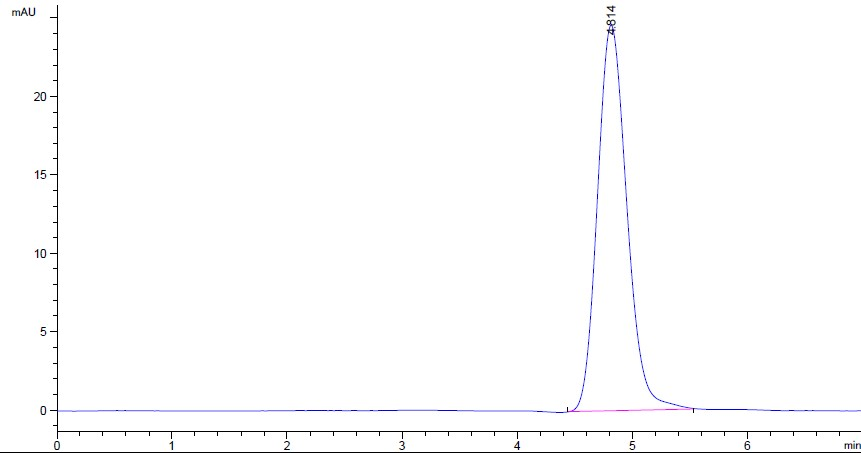


**Supplementary file S2** The chromatogram of nitrate analysis for tomato fruit.
